# Novel object recognition in *Octopus maya*

**DOI:** 10.1007/s10071-023-01753-6

**Published:** 2023-02-21

**Authors:** Fabian Vergara-Ovalle, Fructuoso Ayala-Guerrero, Carlos Rosas, Hugo Sánchez-Castillo

**Affiliations:** 1 Neuropsychopharmacology Laboratory, Psychology School, Universidad Nacional Autónoma de México, colonia copilco universidad, Avenida Universidad 3000, 1er Piso Edif. B. Cub B001, Alcaldía de Coyoacan, Ciudad Universitaria, Coyoacán, CP 04510 Mexico City, Mexico; 2 Neurosciences Laboratory, Psychology School, Universidad Nacional Autónoma de México, Ciudad Universitaria, Coyoacán, 04510 Mexico City, Mexico; 3 Applied Ecophysiology Laboratory, Facultad de Ciencias, Unidad Multidisciplinaria para Investigación y Educación, Universidad Nacional Autónoma de México, Puerto de abrigo s/n Sisal, Yucatán, Mexico

**Keywords:** Exploration, Memory, Novel object, Octopus, Recognition, Behavior

## Abstract

The Novel Object Recognition task (NOR) is widely used to study vertebrates' memory. It has been proposed as an adequate model for studying memory in different taxonomic groups, allowing similar and comparable results. Although in cephalopods, several research reports could indicate that they recognize objects in their environment, it has not been tested as an experimental paradigm that allows studying different memory phases. This study shows that two-month-old and older *Octopus maya* subjects can differentiate between a new object and a known one, but one-month-old subjects cannot. Furthermore, we observed that octopuses use vision and tactile exploration of new objects to achieve object recognition, while familiar objects only need to be explored visually. To our knowledge, this is the first time showing an invertebrate performing the NOR task similarly to how it is performed in vertebrates. These results establish a guide to studying object recognition memory in octopuses and the ontological development of that memory.

**Supplementary Information:**

The online version contains supplementary material available at 10.1007/s10071-023-01753-6.

## Introduction

Cephalopods show a wide variety of behaviors; these behaviors manifest cognitive capacities such as learning (Fiorito and Scotto 1992; Tomita and Aoki 2014; Bublitz et al. 2017), emotions (Kuba et al. 2006), puzzle-solving (Richter et al. 2016) and individual recognition (Tricarico et al. 2011). Different taxonomic groups share these capacities: insects (Simons and Tibbetts 2019), crustaceans, arachnids, and vertebrates (Roth 2013). It is important to note that, the nervous system of every one of these groups differs from each other, with more than 550 million years of evolutionary history; therefore, having these cognitive capacities in each one of them most likely reflects an evolutionary convergence. This convergence has attracted various research groups' attention to understanding how cephalopods achieve these capacities, mainly memory. One of the strategies to study this is by comparing cephalopods and vertebrates (Shigeno et al. 2018). Still, beyond this, the study of memory in cephalopods may help us understand the conditions and constraints required to achieve different types of memory in a nervous system utterly different from that of vertebrates, understanding memory as a cognitive capacity that arises from neural networks, regardless of its evolutionary history.

To achieve this, it is necessary to establish a task that different taxa can perform, which may be evaluated similarly in each of them. A good candidate for this is the novel object recognition (NOR) task (Blaser and Heyser 2015). This task is based on the innate behavior of animals when encountering a new object in a familiar environment; the response can be exploration or aversion. The NOR task consists of an indirect evaluation of memory through discrimination based on familiarization. Also, this task has several characteristics that make it suitable for studying the neurobiology of memory; for example, it does not require conditioning, has high ecological validity, is performed quickly, it can be divided into phases (acquisition, consolidation, and retrieval), as well as temporality (long and short-term memory) and finally it has been observed that it is an ability shared by all groups of vertebrates and some invertebrates could share it; However, it still needs to be confirmed experimentally (Blaser and Heyser 2015). This task has been widely used in vertebrates, for example, in fish such as sharks and zebrafish (May et al. 2016; Toms and Echeverria 2014; Fuss et al. 2014); birds such as pigeons (Spetch et al. 2006), crows (Stöwe et al. 2006) and mammals such as Rhesus monkeys (Rajalingham et al. 2015), mice (Leger et al. 2013) and rats (Mathiasen and DiCamillo 2010). Conversely, NOR has been poorly studied in invertebrates, some similar tests have been carried out in cuttlefishes and insects, like object discrimination and context discrimination (Billard et al. 2020; Solvi et al. 2020; Kelman et al. 2008), but not in the same way as the NOR task in vertebrates, so there is still much research to be done in the field.

Although in octopuses, the possibility of remembering objects or places has been anecdotally and experimentally described (Mather 1991), so far, experimental tasks like the NOR task have yet to be standardized. Beyond that, octopuses spend weeks or months in the same den and feed by foraging. Hence, it would be reasonable to hypothesize that octopuses can discriminate between a familiar and a novel object. That is why in this study, our aim was to standardize this task in *Octopus maya* (Voss and Solis 1966) at different ages, to obtain a method for the complete evaluation of novel object recognition memory in this octopus species and at the same time offer a standardized task that allows applying NOR task in other cephalopod species.

## Materials and methods

### Ethical statement

All the experiments and handling were carried out under the approval of the bioethics committee of the Faculty of Psychology, UNAM, and in accordance with the directive 2010/63/EU (European Parliament), considering the recommendations by Fiorito et al. 2015.

### Subjects

The subjects were obtained from the Applied Ecophysiology Laboratory of the UMDI, SISAL. *O. maya* subjects of three different ages were used; a group of five subjects four weeks post-hatching (“babies”), another group of five subjects four months old (“juvenile”), and, the last group of five subjects of unknown age but weighing more than 800 g, for which were animals in the reproductive stage (“adults”). The babies and juvenile subjects were raised and maintained in laboratory conditions until the end of the experiments, while the adult group consisted of captured subjects. The animals were kept in 125 L tanks (50 cm length, 50 cm width, 50 cm height). Juvenile and adult groups were housed individually, and babies were kept all five together in the same housing tank.

### Animal maintenance

Octopuses were maintained in artificial seawater (salinity 3.5%, pH 8, Nitrite 0, Nitrate 25, Ammonia 0, O_2_ > 95%) on a 12–12 h light cycle with white and red led lights. The experimental tanks or arenas used during the task differ between groups due to the significant difference in size that the octopuses present during their different stages of development. The group of babies carried out the task in 2 L (L) opaque plastic containers, (20 cm length, 10 cm width, 10 cm height); for the juvenile group, 60 L (40 cm length, 40 cm width, 38 cm height) glass containers were used, covered by opaque plastic on three sides; finally, for the adult group, 150 L (60 cm length, 50 cm width, 50 cm height) glass tanks were used, covered by opaque plastic on three sides. To avoid the subjects' stress during the whole experiment, a den was placed in each housing and experimental tank. The den was placed on the side of the tank, centered. This den must guard the animal against the light and be of a suitable size so that the entire octopus can fit inside, and all eight arms can always be touching the internal surface of the den. The use of this den increases the willingness of the animal s to explore the objects that will be kept for them in the following phases.

### Novel object recognition task

The NOR task was adapted from other animal models (Lupetow 2017; Ennaceur 2018; Rossato et al. 2019), with modifications according to the octopus’s behavior.

The task consisted of three phases: habituation, familiarization, and test. The three phases were conducted consecutively inside the experimental tank. During the habituation phase, the specimen was placed into the arena and allowed to get used to the environment for 24 h. Previously, our laboratory group has standardized that *O. maya* spends more time resting in its den than crawling or climbing when familiar with the environment (data not shown). For this reason, it was established that spending more than two continuous hours in its den during the 24 h of habituation would be a criterion to continue to the next phase.

The familiarization phase began at the end of the habituation phase. During this phase, two identical objects were presented; the objects were approximately the size of the mantle of the subject. Because we observed that a bigger object would provoke aversion, and a smaller object could be ignored. Since the mantle length varied considerably for each experimental group (babies: 1.1 ± 0.3 cm, juveniles: 7.4 ± 0.6, adults: 24.8 ± 3.2 cm), acrylic figures of different sizes were used (supplementary Fig. 1). These objects were placed at the same distance from the den and on opposite sides. The objects were presented when the subject was inside or very close to its den. This phase lasted 30 min. This time was standardized to increase the chance that the subjects would explore each of the two objects for at least 20 s, as suggested for other taxa (Ennaceur 2018; Rossato et al. 2019).

**Fig. 1:**
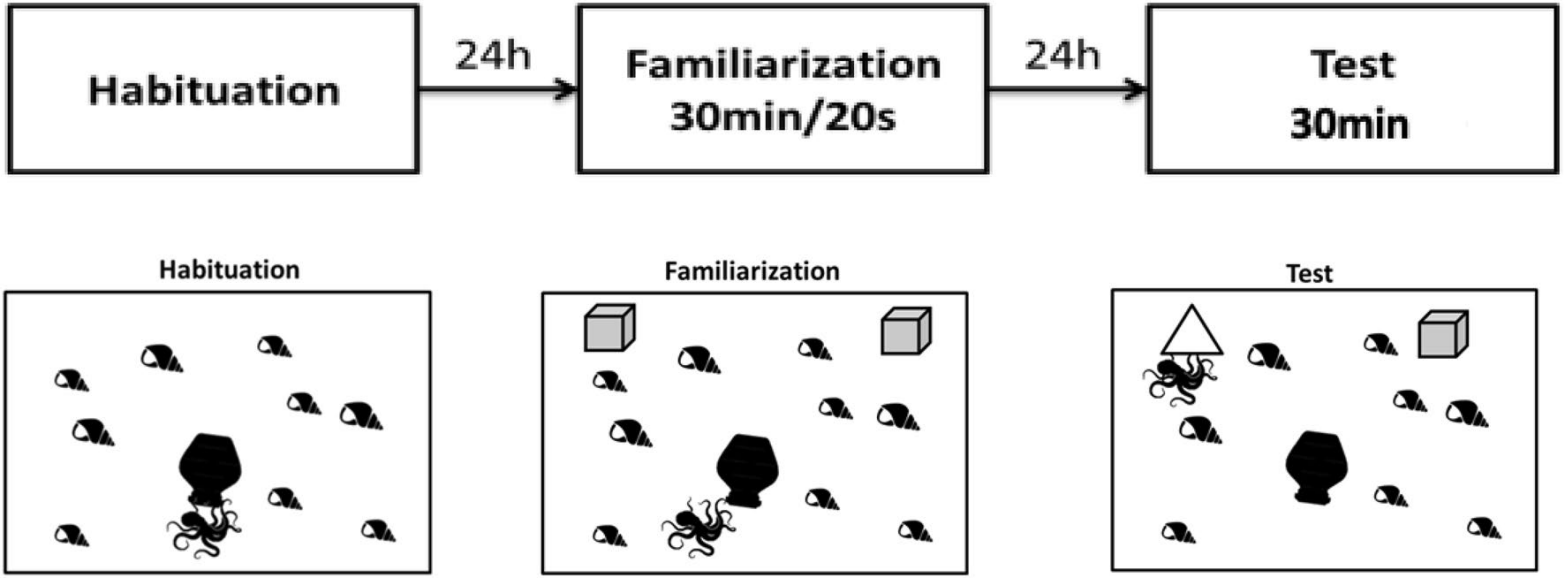
Experimental diagram of the novel object recognition task for *O. maya.* During familiarization, the stage of memory acquisition occurs, while in the test phase, the evocation of memory is observed

The testing phase started at the end of the familiarization phase. During this phase, one of the objects was presented the day before, and a new one with different characteristics of shape and color was given. Familiar and new objects identities were counterbalanced between individuals. Opaque and transparent objects were used, with blue, red, and white colors. No difference was found in test results, regardless of the colors used. This phase lasted 30 min, regardless of how long they explored each object. During the familiarization and test phases, the total exploration time and the visual and tactile exploration of the objects were quantified. The visual exploration consisted of indirectly approaching one of the objects, with climbs or jumps, and with at least one of the eyes directed toward the object. These movements can continue until they get as close to the object as possible but without touching it. The tactile exploration phase is when the specimen touches the object with one or more arms, considering that these objects were not attached to the tank walls; hence, the subjects could lift or push them. (Fig. 1).

Due to the test duration, the subjects were fed at the end of each day as part of their maintenance in captivity. In addition, octopuses with high levels of stress tend to reject food (Fiorito et al. 2015); therefore, as a way of corroborating that the animals were not stressed and could continue with the test, at the end of each phase they were fed (shrimp) and only those subjects that accepted food at the first attempt, could continue to the next phase. As a result, all the subjects took the food on the first feeding attempt.

### Statistical analysis

The data were evaluated using the discrimination index ((Novel-Familiar)/Total exploration) and the non-parametric Mann–Whitney *U* test to compare the exploration between groups and Wilcoxon signed-rank test with the software STATISTICA 10 to compare the exploration time of the familiar object with the novel one, considering *p* < 0.05 as statistically significant.

## Results

### When juveniles or adults,* O. maya* differentially explores a known and new object

**Fig. 2:**
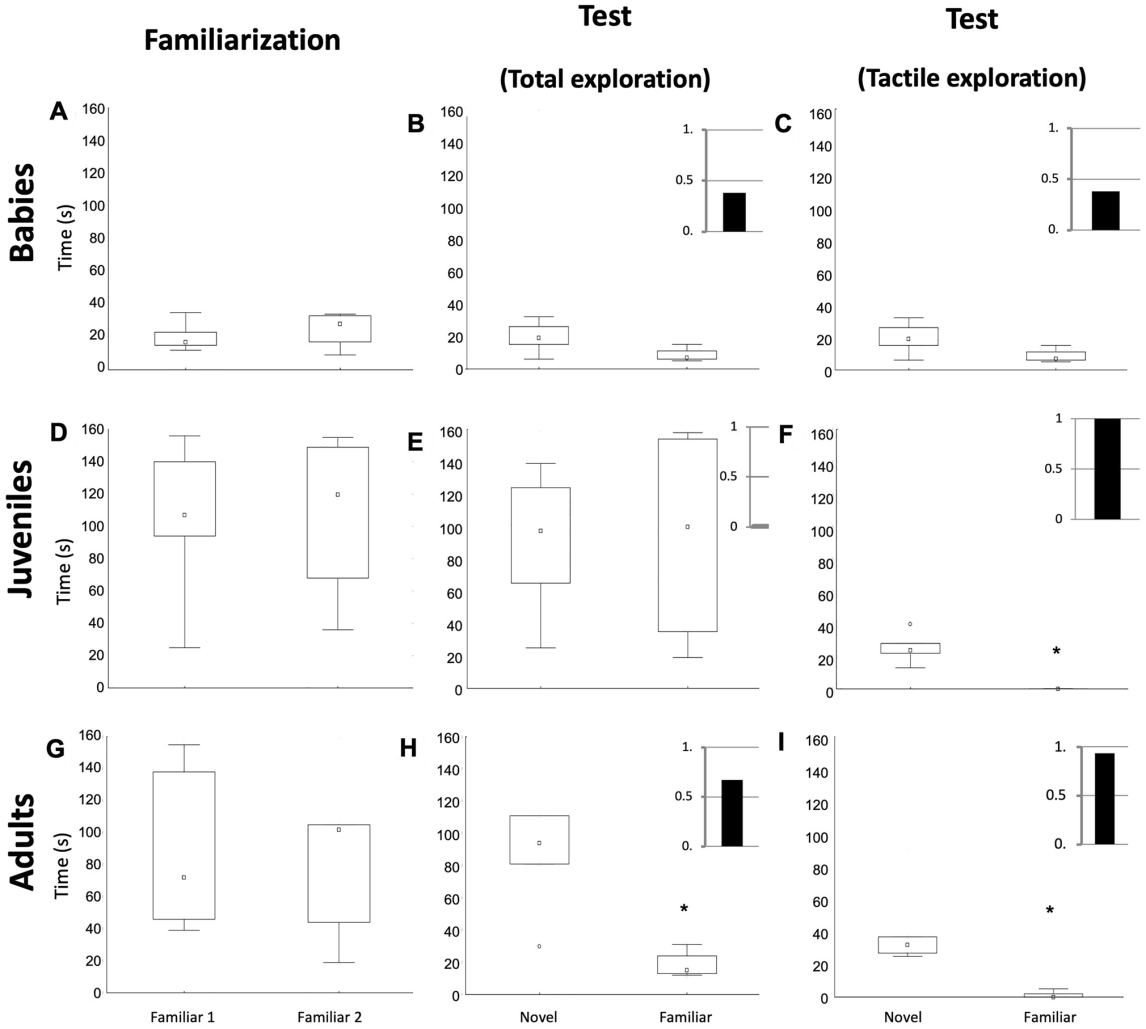
Results of the NOR test in babies (**A**–**C**), juveniles (**D**–**F**), and adults (**G–**I) of *O. maya*. During the familiarization phase (**A**, **D**, and **G**), there are no differences between the total exploration time of both objects. However, it is observed that babies explored for less than 60 s, while the juvenile and adult groups explored for more than 100 s. In the total exploration during the test phase (**B**, **E**, **H**), only adults (**H**) show a significant difference (Wilcoxon, *p* value = 0.041, *Z*: 2.03). If only tactile exploration is considered, both juvenile and adult groups showed a differential behavior between the novel and familiar object (Wilcoxon, *p* value = 0.043, *Z*: 2.02 and Wilcoxon, *p* value = 0.028, *Z*: 2.2; respectively). The insets on each test graph show the discrimination ratio value (DR) = (Time exploring the novel object – Time exploring the familiar object) / Time of total exploration). A DR close to 1 is considered a good discrimination between the familiar object and the novel one, while a DR ≤ of 0.5 is considered an inability to distinguish between the familiar object and the novel one (Ennanceur 2018; Sivakumaran et al. 2018)

The juvenile and adult groups showed similar behavior with two types of object exploration; visual and tactile guided exploration. After the objects were explored, the animals returned to their dens in a resting position (Supplementary Fig. 2). In the juvenile and adult groups, it was observed that the exploration times were similar between both objects during familiarization (Mann–Whitney *U* test, *p* value = 0.28, *Z* = 1.088). Although the time dedicated to each type of exploration could vary, the five subjects presented an approach and manipulation of both objects (Fig. 2A). After 24 h, the test phase was carried out, and it was observed that the adult subjects explored the familiar object for less time, compared to the novel object (Wilcoxon, *p* value  =  .043, *Z*: 2.023) (Fig. 2B), while the juvenile explored the novel object in a tactile way for a significantly less time (Wilcoxon, *p* value = 0.028, *Z*: 2.2), but they showed the same visual exploration time (Wilcoxon,* p* value = 0.23, Z*:* 0.94). Interestingly, it was observed that there was no manipulation when exploring the familiar object; when the object is known, only visual exploration is used for its identification, but not tactile exploration (Fig. 2C).

### At four weeks old,* O. maya* does not behave differentially concerning the novel object and the familiar one

**Fig. 3:**
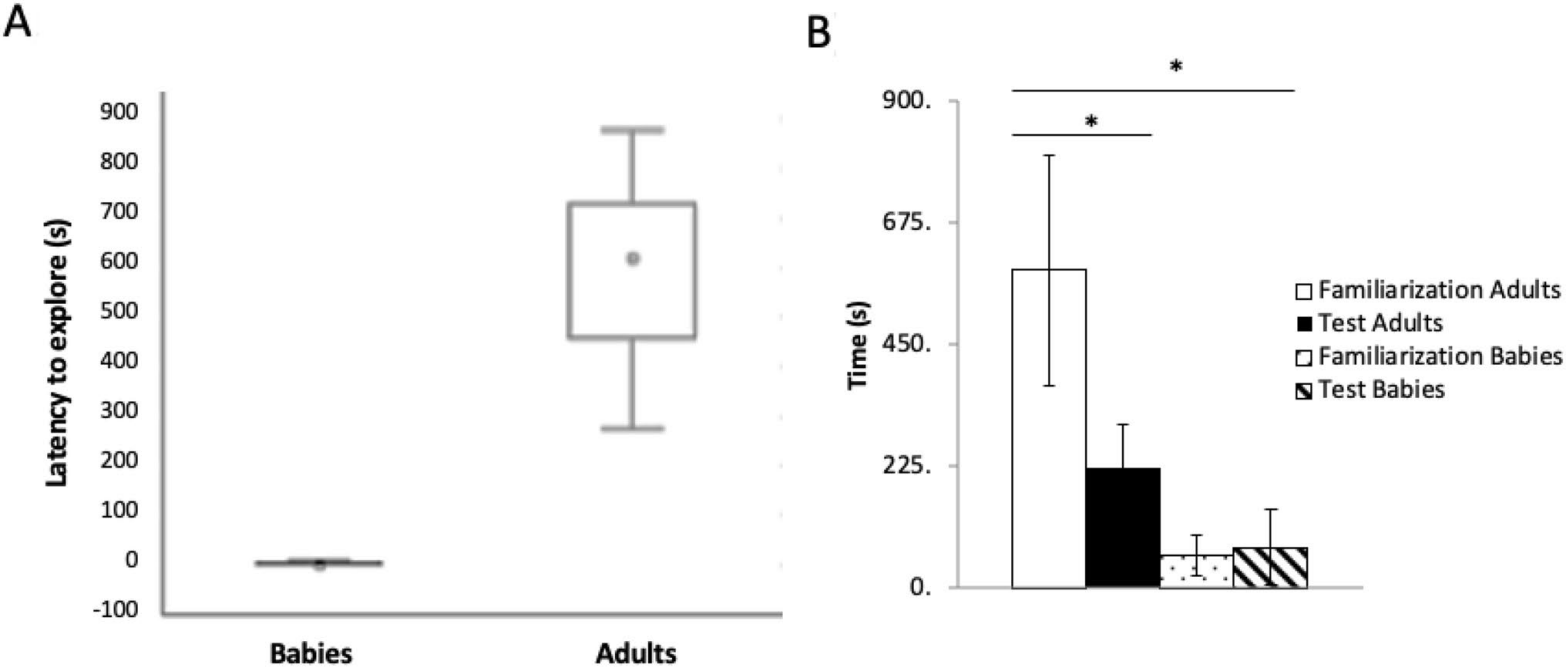
Latency to explore (**A**) and total exploration time during the NOR test in “adult” and “baby” groups (**B**). The whole exploration time in adults was shorter during the test phase (Wilcoxon, *p* value = 0.043, *Z*: 2.02). The babies group explored significantly less time than the adults (Wilcoxon, *p* value = 0.021, *Z*: 2.3); however, they did not show differences between the familiarization and test phases

When conducting the baby group test, an immediate “attack” behavior toward the objects was observed, meaning the subjects pounced on them immediately as they entered the water. This reflected in the decrease of start latency during the test phase (Fig. 3A). This immediate attack behavior seems to occur regardless of the size of the objects, since the subjects presented it toward the familiar and novel objects but also toward a net (10 cm), a sponge (7 cm) and even the hand of the experimenter during the cleaning of the tank. For this reason, there was no division regarding the exploration phases; only the time in tactile exploration and the total exploration time (the time spent outside the den or in an activity other than the rest) were evaluated. In these subjects, it was observed that although there is an increase in the time they spend in the tactile exploration of the novel object, compared to the familiar object, this difference did not occur in all individuals and is not statistically significant (Wilcoxon, *p* value = 0.068,* Z*: 1.82) (Fig. 2E). On the other hand, they spent significantly less time exploring than the adult group (Fig. 3B).

## Discussion

The novel object recognition task is a simple and short-term task that has been proposed as ideal for studying and comparing the memory of different groups of animals. However, it must be adapted to the biology of each group (Rosas et al. 2014), considering behavior, the time between phases, the characteristics of the objects, and the arena. The present study observed that juvenile and adult subjects of *O. maya* showed novel object recognition. This recognition is observed as a decrease in the total exploration time of the familiar object. The exploration can, at least, be visual and tactile. We observed that when the object becomes familiar, it is practically not explored tactilely. The results suggest that the familiar object can be recognized visually without the need to explore it with another sensorial sense. On the other hand, when the object is novel, the two most prominent senses of the species are used to explore it.

Unlike what was observed in the juvenile and adult groups, most of the animals in the babies’ group did not show adequate recognition of a novel object. Instead, they showed an immediate attack behavior toward both objects. We propose that this immediate attack behavior could have an ecological explanation since getting food can be complicated in the octopus’s early stages of life. In such a way that the priority is to attack and try to eat anything that comes nearby them. On the other hand, juvenile and adult subjects could prioritize protecting themselves from possible predators rather than searching for food; therefore, they achieve a better inhibition of the attack behavior, allowing them to take the time to discriminate novel objects from familiar ones. Also, we hypothesize that at the babies stage, tactile exploration could be more important, rather than visual exploration because of the behavior of individuals to pounce on objects with all eight arms, in a similar way to the attack behavior reported for other species of octopuses, for example, *O. vulgaris* (Shomrat et al. 2008; Zarrella et al. 2015).

These results suggest that *O. maya* goes through a maturation stage, during which more complex responses are acquired, allowing better discrimination with the objects to be explored. This should be reflected in the post-hatching nervous system maturation of the species (Vergara-Ovalle et al. 2022). Something similar was found by Anderson et al. 2004 during NOR in rats. As in our work, they also used three groups of different ages: weanling, juveniles, and adults. They observed that juveniles and adult rats can discriminate between a novel and a familiar object, 24 h after familiarization, whereas weanling rats could not. This seems to indicate a process of nervous system maturation, suggesting a similarity between *O. maya* and rats. It is important to mention that to achieve an adequate exploration of the rats of different ages during the task, Anderson et al. made the task age appropriate by downsizing the objects and the arena similarly to how it was done with *O. maya* in the present work. This ontological difference during the NOR task seems not to be universal; for example, zebrafish larvae can discriminate objects from the first days after fertilization (Bruzzone et al. 2020). Perhaps this difference in the ability to discriminate from the earliest stages of life corresponds to the need to mature a more complex nervous system, considering that zebrafish have 10^7^ neurons (Friedrich et al. 2013), while rats have an accelerated increase in the number of neurons during the first months after birth and reach 2 × 10^8^ neurons (Bandeira et al. 2009). Similarly, octopuses considerably increase their number of neurons during post-hatching development to reach 5 × 10^8^ neurons (Hochner 2012).

As far as we know, this is the first time this task has been used in a species of octopus, and similar adaptations have only been made in a few invertebrates such as cuttlefish (Kelman et al. 2008; Billard 2020) and bumblebees (Solvi et al. 2020). However, these were not strictly NOR tasks but adaptations or another type of discrimination. Here, octopuses were tested with a similar task performed in vertebrates, thus facilitating the comparison between both at a behavioral and cognitive level. Conversely, the effect that "familiarity of context and objects" has on learning in some species of octopus has been widely described (Fiorito et al. 1998; Borrelli et al. 2020), however, to our knowledge, it had not been standardized in a task that could be compared with other groups and measurable as a memory test, until now.

Also, *O. maya* presents an innate exploration behavior to novel objects, in a similar way to what happens in murine models (Ennaceur 2018), but different from what happens in other vertebrates such as Danios, who have an aversion to novel objects or neophobia (Fuss et al. 2014). Because juvenile and adult *O. maya* subjects tend to move slowly, the duration of the different phases was longer than normally used in mice and rats. In this work, the duration of each phase of 30 min is recommended for *O. maya*, while it varies between 1 and 15 min in mammals. (Antunes and Biala 2012). Another relevant difference between rats and octopuses is the presence of a den during the test, which is used to avoid the subjects' stress and achieve correct habituation. Though this is an important difference between the task in the murine model and octopuses, it does not interfere with the NOR task since it is part of the context throughout the test. Regarding the exploration time, the adult subjects total exploration time decreased significantly during the test phase. Even though in the murine model, this would normally be considered as a problem of lack of motivation or motor skills during the test phase, in *O. maya,* it can be explained considering that the subjects returned to their resting position in the den once the exploration of the objects was completed. During the test phase, the familiar object was not always explored since the total exploration time was reduced because the animals almost only invested time exploring the novel object.

In vertebrates, NOR has been associated with the activity of the hippocampus, insular cortex, perirhinal cortex, and medial prefrontal cortex (Tanimizu et al. 2017; Rossato et al. 2019; Cinalli et al. 2020). Although no invertebrate has such structures, including octopuses like *O. maya*, this and probably other octopuses species show an evolutive convergence with vertebrates, the ability to remember a familiar object. Therefore, some structures in the central brain of octopuses have been related to memory tasks, and similarity has been sought between these and the structures of the vertebrate brain, particularly its vertical lobe has been compared with the hippocampus of vertebrates (Shomrat et al. 2015; Shigeno and Ragsdale 2015; Shigeno et al. 2018). While this comparison helps to understand what happens in the octopus brain, from our knowledge of other more studied groups, it is important to consider a complete system rather than a similarity for each structure.

To integrate the knowledge of how the octopus brain works, studies were made, dividing the supraesophageal region of the octopus brain into two systems; one that includes the vertical lobe and the superior frontal lobe (VL-SF) responsible for visual memory tasks and another that involves the buccal lobe and the inferior frontal lobe (Bu-IF), responsible for somatosensory memory tasks (Wells and Young 1975). This division is useful when evaluating tasks such as visual discrimination (Sutherland 1962; Tomita et al. 2014) or fear of condition (Shomrat et al. 2008); however, in the results of this work, it is clear that in the NOR task, *O. maya* uses visual and tactile exploration. Hence, we propose that this task might involve both VL-SF and Bu-IF systems. This is supported by the histological observations in some octopuses’ brain atlas (Jung et al. 2018; Vergara-Ovalle et al. 2022), as there is a neuronal connection between the superior frontal lobe and the inferior frontal lobe. This might conform to a physical substrate for the connectivity of the possible systems required for *O. maya* to remember the familiar object. It is giving rise to propose a series of experiments that allows corroboration of whether these systems participate and the role of the brain's different structures during the NOR. It would be interesting to see if visual exploration is essential for NOR in *O. maya* since this study shows that once the octopus becomes familiar with the objects, it does almost not require tactile exploration to recognize them.

The present study gives evidence of evolutionary convergence between vertebrates and *O. maya* that allows the recognition of novel objects in the environment. Essentially, this task can be evaluated almost identically between both groups and opens the opportunity to compare the physiological processes that underlie it, such as neuronal plasticity, protein, and RNA synthesis, the participation of different transcription factors, and systems of brain structures that compose it, among others.

## Supplementary Information

Below is the link to the electronic supplementary material.

Supplementary file1 (DOCX 1054 KB)

## Data Availability

All data are available from the corresponding authors upon reasonable request.
