# Supplementary material for: Novel object recognition in Octopus maya
Source: Anim Cogn. 2023 Feb 21;26(3):1065–72. doi: 10.1007/s10071-023-01753-6 (PMC10066149; doi:10.1007/s10071-023-01753-6)
Supplement: Supplementary file 1 — Supplementary file1 (DOCX 1054 KB) [file 10071_2023_1753_MOESM1_ESM.docx]

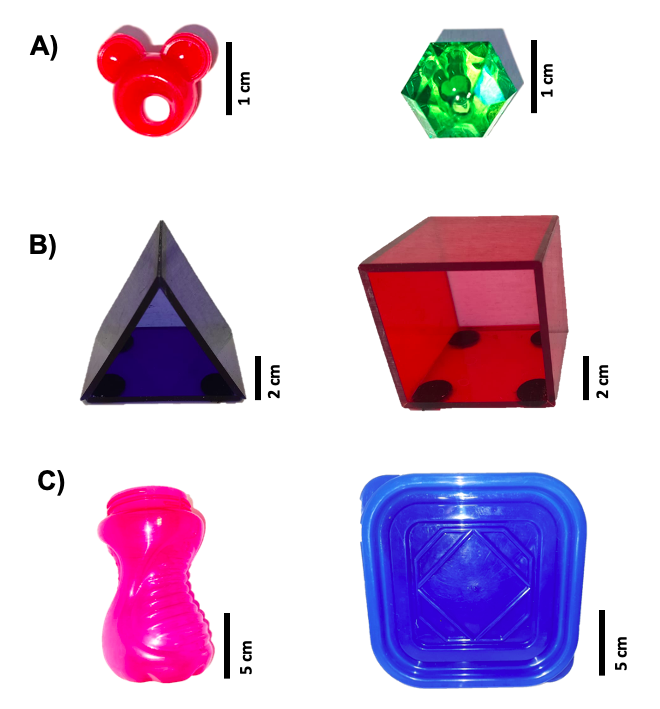


**Supplementary figure 1. Examples of objects used for testing.** The size of these objects changed depending on the average mantle size in each group; A) babies 1.5cm, B) juveniles 6.5cm, and C) adults 18 cm. Simple acrylic figures were used, and their identity (new or familiar) was counterbalanced between individuals.


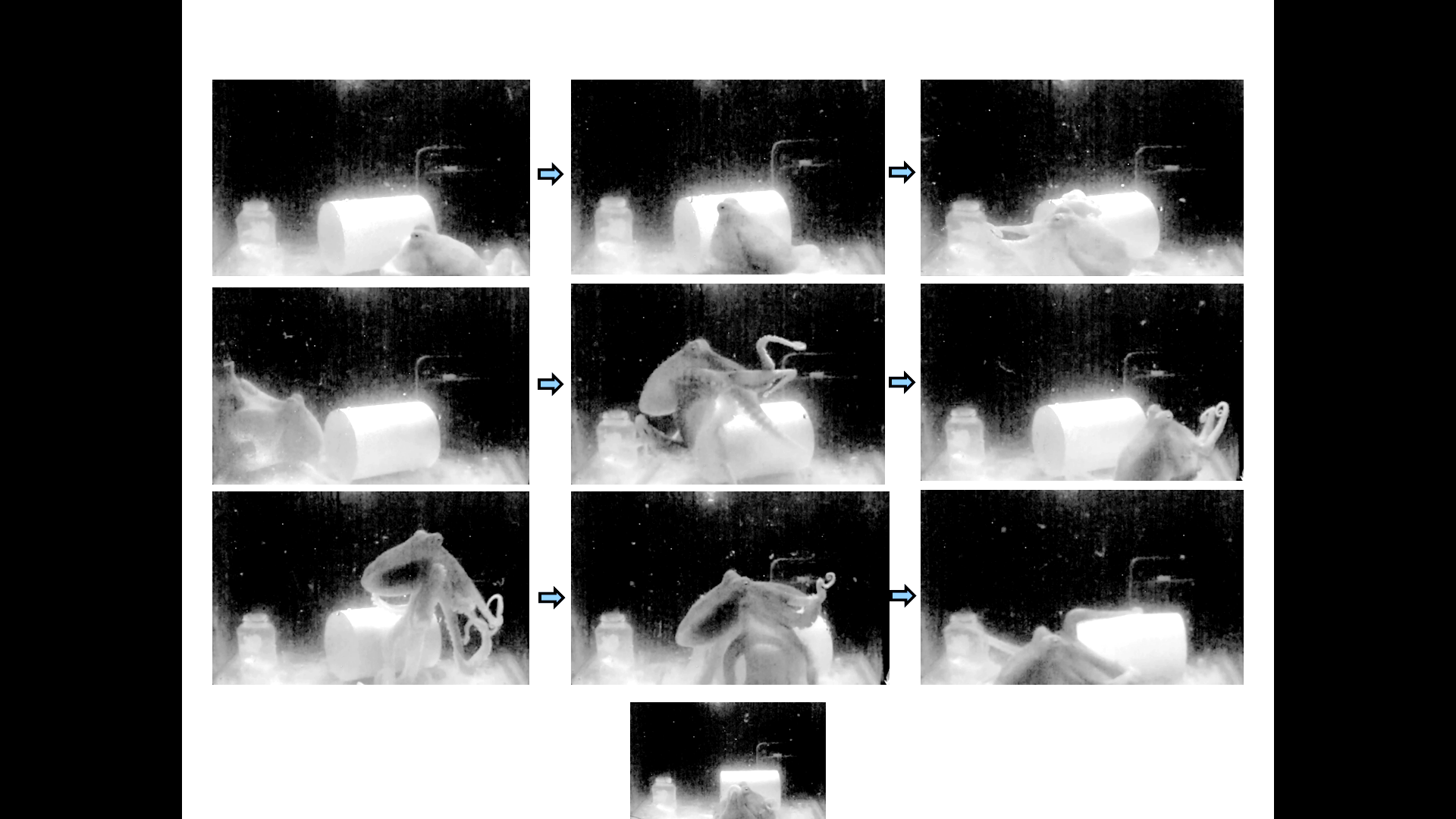


**Supplementary figure 2.** When presented with a novel object (left of the octopus), octopuses tended to direct both eyes toward the object, increase climbing and touch the novel object with at least two arms. Instead, when presented with a familiar object (right of the octopus), octopuses usually avoid touching it and explore it only visually.
